# Bronchiolitis Simulation Module in the Pediatric Preclerkship Educational Exercises (PRECEDE) Curriculum

**DOI:** 10.15766/mep_2374-8265.11318

**Published:** 2023-06-13

**Authors:** Justin M. Jeffers, Amit Pahwa, Stacy Cooper, Olivia Widger, David W. Cooke, Emily Frosch, Rebekah Reisig, Christopher Grybauskas, Eric Balighian, Lauren Kahl, Edward L. Bartlett, W. Christopher Golden

**Affiliations:** 1 Assistant Professor, Department of Pediatrics, Johns Hopkins University School of Medicine; Adjunct Faculty, Johns Hopkins University School of Education; 2 Associate Professor, Department of Pediatrics, and Associate Professor, Division of General Internal Medicine, Johns Hopkins University School of Medicine; 3 Assistant Professor, Department of Pediatrics, Johns Hopkins University School of Medicine; 4 Clinical Assistant Professor of Pediatrics, Perelman School of Medicine at the University of Pennsylvania; 5 Associate Professor, Department of Pediatrics, Johns Hopkins University School of Medicine; 6 Associate Professor, Department of Psychiatry and Behavioral Sciences, Johns Hopkins University School of Medicine; 7 Program Coordinator, Department of Pediatrics, Johns Hopkins University School of Medicine; 8 Clinical Associate of Pediatrics, Johns Hopkins University School of Medicine

**Keywords:** Bronchiolitis, Pediatric Emergency Medicine, Pediatrics, Respiratory Therapy, Simulation

## Abstract

**Introduction:**

Acute bronchiolitis is a viral infection infecting 90% of children under the age of 2 years, with approximately 200,000 deaths per year. The current standard of care remains largely respiratory support and prevention. Therefore, understanding how to assess and escalate respiratory supportive care is paramount for health care providers taking care of children.

**Methods:**

We used a high-fidelity simulator to simulate an infant with progressing respiratory distress in the setting of acute bronchiolitis. The participants were pediatric clerkship medical students during their preclerkship educational exercises (PRECEDE). The students were asked to evaluate and treat the simulated patient. After debriefing, the students repeated the simulation. We assessed both performances via a weighted checklist specifically developed for this case to measure team performance. Students also completed an overall course evaluation.

**Results:**

Ninety out of 121 pediatric clerkship students were enrolled. Performance improved from 57% to 86% ( *p* < .05). Donning appropriate personal protection equipment was the most missed item both pre- and postdebriefing. Overall, the course was well liked and received. Participants requested more simulation opportunities within PRECEDE as well as a summary document to reinforce learning.

**Discussion:**

Pediatric clerkship students improved their performance managing progressing respiratory distress due to acute bronchiolitis via a performance-based assessment tool with sound validity evidence. Improvements going forward include improving faculty diversity and offering more simulation opportunities.

## Educational Objectives

By the end of this simulation, participants will be able to:

1. Recognize respiratory distress as evaluated by an evaluation checklist.
2. Utilize the ABC (airway, breathing, circulation) assessment strategy and crisis resource management principles.
3. Describe an age-appropriate differential diagnosis for infant respiratory distress.
4. Implement a stepwise approach to treating infant respiratory distress using various oxygen delivery systems.
5. Describe the indications for diagnostic tests such as imaging and labs.

## Introduction

Acute bronchiolitis is a disease process resulting in airway inflammation and obstruction of the small bronchioles.^1^ It is almost exclusively caused by viral infections in children less than 2 years of age^2^ and is one of the most substantial health burdens worldwide for this age group.^1^ Globally, there are approximately 3.4 million hospital admissions and about 200,000 deaths per year, with over 99% of deaths occurring in developing countries.^3^ This makes acute bronchiolitis the most common cause of hospitalization for children under 12 months.^4^

Respiratory syncytial virus infects 90% of children under 2 years of age^4^ and, along with rhinovirus, accounts for 50%-80% of hospitalized cases of bronchiolitis, with coinfection common.^5^ Acute bronchiolitis typically occurs between fall and spring each year, peaking during winter months.^6^ The typical disease course lasts 7–10 days; symptoms ramp up and peak between days 3 and 5, with most cases managed at home with mild upper respiratory symptoms.^1,2^ However, the overall risk of recurrent wheezing and asthma postinfection is 50% through school-age years and may persist into adulthood, resulting in further burden of care.^1^

Given the large burden of disease, numerous treatment options have been studied, with little to no benefit. The most recent American Academy of Pediatrics clinical practice guideline recommends that clinicians should not administer beta-agonists, epinephrine, hypertonic saline in the emergency department, systemic corticosteroids, or supplemental oxygen if the oxyhemoglobin saturation exceeds 90%.^4^ Chest physiotherapy and antibiotics are also not recommended.^4^

Therefore, treatment of bronchiolitis remains supportive, focusing on respiratory support with oxygen, nasal suctioning, positive pressure, and hydration status.^2^ Prevention remains the most valuable tool in the control of acute bronchiolitis. There is a vaccine available, palivizumab, but its use is limited to patients in their first year of life with hemodynamically significant heart disease or patients with chronic lung disease of prematurity.^4^ Handwashing with soap and water, as well as alcohol-based rubs, is effective at preventing the spread of viruses that cause bronchiolitis.^4^ Because treatment is largely supportive, understanding how to escalate respiratory supportive care is paramount for all pediatric providers.

For such a common pediatric disease process, simulation-based learning (SBL) curricula in the literature are limited. A search of *MedEdPORTAL* revealed two stand-alone SBL cases^7,8^ and three SBL cases as part of a larger curriculum.^9–11^ Some of the cases were developed prior to the most recent treatment guidelines.^7,9–11^ Most have a limited or no assessment and evaluation strategy; those that do primarily focus on confidence and knowledge.^9–11^ None of the published curricula have evaluation tools that assess performance.

The purpose of our SBL is to provide pediatric clerkship students the opportunity to evaluate and manage bronchiolitis and infant respiratory distress focusing on the escalation of respiratory supportive care according to current guidelines. We also share a performance-based assessment tool with sound validity evidence.^12^

## Methods

### Development

This simulation was developed as part of the Johns Hopkins University School of Medicine pediatric preclerkship educational exercises (PRECEDE) curriculum.^13–16^ The faculty who developed the simulation were experts in pediatrics, pediatric emergency medicine, medical education, SBL, neonatology, pediatric hospital medicine, pediatric oncology, and pediatric psychiatry. They had an average of 13 years of clinical experience, and most were medical educators/simulationists. The simulation was performed in the Johns Hopkins University Medical Simulation Center.

Pediatric clerkship students participated in the simulation during the Mid-Cede portion of PRECEDE, a 2-day curriculum occurring in the middle of the pediatric clerkship. No presimulation readings or assignments were given. However, earlier PRECEDE modules focusing on pediatric age-specific vital signs, interpretation of pediatric-specific laboratory data,^14^ and critical thinking skills provided a foundation for this module. Additionally, clerkship students received Advanced Cardiac Life Support training as part of a Transition to the Wards curriculum that bridged their primarily nonclinical and clinical years of medical school education.

### Equipment

This simulation case used the following equipment, with reasonable alternatives in parentheses:

• High-fidelity infant simulator capable of simulating infant respiratory distress (a low-fidelity simulator could be used)
• Monitor with capability to display heart rate, respiratory rate, pulse oximetry, and blood pressure (vital signs could be verbalized if no monitor)
• Patient leads to measure heart rate, respiratory rate, pulse oximetry, and noninvasive blood pressure (lead attachment could be verbalized when asked by learner if no leads are available)
• Infant car seat or infant chair
• Suction (nasal and off the wall)
• Crash cart or stand-alone emergency equipment
  ○ Age-appropriate nasal canula, nonrebreather (NRB), bag-mask ventilator (BMV), and oral and nasal airways
  ○ Intravenous access and/or intraosseous device equipment
  ○ Medications, including normal saline bolus, maintenance intravenous fluids, albuterol, racemic epinephrine, and steroids
  ○ Defibrillator with age-appropriate pads (not mandatory since the patient does not go into cardiac arrest; however, we encourage defibrillator placement for all acutely decompensating patients)
  ○ Age-appropriate syringes and needles

### Personnel

Pediatric clerkship students at the midpoint of their pediatrics clerkship participated in this simulation in groups of four to five. Two physician faculty facilitators were present for each group. The first faculty member served as a nurse embedded participant. The second faculty member served as the simulation technician and was responsible for adjusting the manikin response appropriately. For institutions with simulation technicians available, the second faculty facilitator can participate in other ways (e.g., as a parent). Both faculty facilitators participated in the debriefing of the simulation. Participants consented to participate and to video/audio recording.

Depending on an institution's resources, personnel can be deployed in a variety of ways. For example, a standardized patient could play the role of a parent, or a nurse faculty member could serve as the nurse embedded participant.

### Implementation

This 1-hour simulation was performed at the Johns Hopkins Medical Simulation Center. Due to the overall group size of 20–24 students, three rooms were used, and the simulation was run twice over 2 hours to accommodate all participants. Half the participants came for the first hour, and the other half came for the second hour. Each room was identically set up based on the equipment list above. As the large group arrived, students were randomly split into smaller groups of four to five and directed to their specific simulation room. Outside the room, a facilitator gave each participant a handout with the case stem and a brief history of the patient (infant admitted overnight with a diagnosis of bronchiolitis) as if the participants were caring for the patient on their inpatient team (Appendix A). No further information or instructions were given. Participants had a few minutes to read the handout while the facilitators performed final checks. Once everything was ready, the in-room facilitator invited the team into the room, and the simulation began. The in-room facilitator wore a communication device to exchange information with the facilitator running the simulator in the operations room. The in-room facilitator was also responsible for prompting participants as needed to move the simulation forward. The case took approximately 15–20 minutes. The debriefing required approximately 25–30 minutes. Then, to reinforce learning, participants repeated the simulation, which took approximately 10 minutes, leaving 5 minutes for a short debriefing and room turnover. The simulation case is presented in detail in Appendix B, and the faculty guide is presented in Appendix C.

### Assessment

A 19-item weighted checklist was developed via a modified Delphi approach yielding content, construct, internal consistency, and response process validity evidence (Appendix D).^12^ Fourteen items involved timed responses. The checklist was divided into three sections: Situational Awareness/General Tasks, Initial Management, and Escalation of Care. Each item included descriptors and was scored as not done, partially or incorrectly done, or done correctly and completely.

The checklist was easy to use, with little to no rater training needed, as shown by the strong interrater reliability and the validity evidence described in the checklist development publication.^12^ A brief rater training to provide an overview and to gain familiarity with the checklist may be beneficial, especially if the simulation case is unfamiliar. Each learner group had one rater (three raters for each session). All faculty were responsible for scoring checklists throughout data collection.

Additionally, overall course evaluations were collected (Appendix E). The course evaluations comprised 10 items. Participants were asked to rate each item as either strongly disagree, disagree, agree, or strongly agree. Where appropriate, this was changed to poor, fair, good, or excellent. The course evaluation also included one open-ended question: “Do you have any recommendations to improve this session? If so, please provide.”

Both the initial simulation and the postdebriefing simulation were scored in real time, and interrater scoring was done via video review. The two were compared using the paired *t* test. Ten percent of the checklists were analyzed for interrater scoring using Cohen's kappa. The course evaluations were analyzed using descriptive statistics. All statistics data were analyzed using Stata 17 (StataCorp).

### Debriefing and Educational Methodologies

The debriefing focused on the objectives of the case as well as the assessment tool scoring. Self-reflection strategies such as advocacy-inquiry^17,18^ and elements of rapid cycle deliberate practice (RCDP)^19,20^ were used.

Examples of frequent questions utilizing advocacy-inquiry included the following:

• “I noticed you left the patient in the infant car seat. I was thinking it would be easier to evaluate the patient on the hospital bed. I'm curious to hear your thoughts about that.”
• “I noticed you started with a nasal canula to deliver oxygen. I was thinking that based on the patient's increased work of breathing and hypoxia, an NRB would be a better choice. Can you share your thought process about that?”
• “You seemed comfortable placing the oral airway adjunct. Would you mind sharing with the group how you did it?”

Although the case itself did not utilize RCDP, aspects of the debriefing did. For example, when teaching a participant how to effectively use a BMV, the facilitator could pause, correct, and allow the participant to try again.

This project was approved as exempt by the Johns Hopkins University Internal Review Board (IRB00148015).

## Results

Ninety out of a possible 121 pediatric clerkship students participated from June 2018 to May 2019 (1 academic year). Seven students declined to participate, and therefore, their entire group of four to five were excluded. Out of 166 total points, assessment tool scoring improved from a mean of 94 (57%, *SD* = 11.5) to 143 (86%, *SD* = 9.6; *p* < .05). The item missed the most was “donning appropriate personal protection equipment (PPE),” with only 3% doing so appropriately across both pre- and postdebriefing. Students showed the greatest improvement on section two of the assessment tool, Initial Management, with scores increasing from a mean of 33 out of 58 (57%, *SD* = 6.1) to 52 (90%, *SD* = 2.2).

In terms of the highest-weighted (most important) items, all six were at least partially done preintervention, and almost all were done correctly postintervention. For example, the item “placed patient on a monitor” had a preintervention mean of 9.2 (*SD* = 1.9) and a postintervention mean of 9.6 (*SD* = 1.4). This performance was similar for all items with a weight of 5 (the highest weight).

Specific items showing the greatest improvement tended to be the lowest-weighted ones (a weight of 3 or 3.5). For example, the item “lowers bed rails” improved from a mean of 1.2 (*SD* = 2.0) to a mean of 5.4 (*SD* = 1.2). Similar improvement was seen for the item “places oral and/or nasal airway.”

Interrater reliability was consistent with that reported in the checklist development process paper.^12^ Overall, the checklist had a Cohen's kappa of 0.81 (95% CI, 0.75-0.88).

Eighty participants (89%) completed the course evaluations (Table 1). Overall, the course was very well received and was rated very highly, with at least 90% rating each question as strongly agree (the highest-possible option). No participants scored any aspect less than agree. Table 2 features representative quotes from the open-ended question. The most common theme for improvement was to offer more opportunities to participate in SBL. The next most common theme was to provide participants with a summary document after case completion.

**Table 1.**
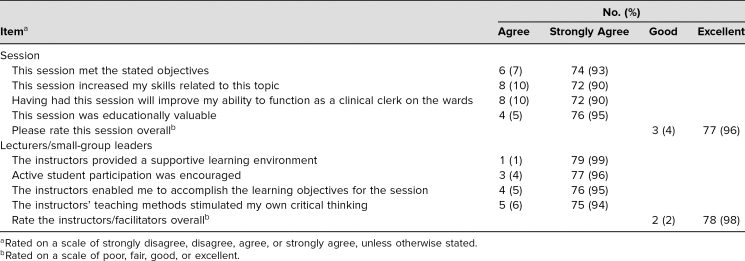
Participants’ Overall Course Feedback (*N* = 80)

**Table 2.**
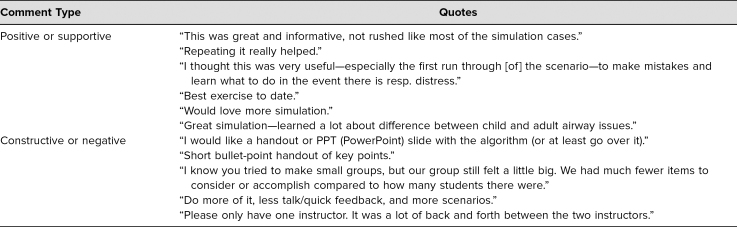
Qualitative Feedback Summary

## Discussion

We developed and implemented an SBL case for pediatric clerkship students focusing on the evaluation and escalation of care for acute bronchiolitis in a hospitalized infant. From 2018 to 2019, 90 participants were enrolled to evaluate this SBL scenario. Overall, there was significant improvement in performance pre- and postdebriefing. Additionally, participants enjoyed the case and were appreciative of the faculty participation and guidance.

To date, this is the only published SBL that both reflects current guidelines^4^ and includes a performance-based assessment tool with sound validity evidence.^12^ Although this case and assessment tool (and its associated validity evidence) were developed specifically for pediatric clerkship students, we believe both can be easily adjusted to fit other learner groups. For example, the continued escalation of respiratory support to include high-flow nasal cannula, continuous positive pressure ventilation, or intubation can be integrated to accommodate more advanced participants.

Donning appropriate PPE was overwhelmingly the most missed assessment tool item. This is important to note due to the contact and droplet nature of bronchiolitis transmission.^1^ However, we believe there was some simulation phenomenon at work, meaning that in a clinical hospital setting, there would have been signage alerting anyone entering the room to wear appropriate PPE. We did not have signage available in our simulation rooms. To make this SBL case more realistic and relevant, we are considering placing signage at the simulation room entrance along with the appropriate PPE.

The most common theme from the open-ended course evaluation question was that the participants wanted more SBL education and experiences. To address this, we are developing a pediatric emergency medicine module for the pediatric PRECEDE curriculum. The module will include this bronchiolitis case as well as an infant nonaccidental trauma case and a septic shock case. Performance-based assessment tools are being developed for these two additional cases via the same modified Delphi approach used for this case. Another common theme was the request for a summary document to be given to learners after the case to reinforce lessons learned. We are considering how to do this in the most educationally effective way.

There are numerous limitations to consider. First is the high-fidelity nature of the simulation. Although this case could be done with a lower-fidelity simulator, we believe the case functions at its best with a high-fidelity simulator. A second limitation is the lack of generalizability of the assessment tool, as the validity evidence collected is specific to this pediatric clerkship group. However, as described, the modified Delphi approach used is a straightforward and relatively low-time and low-cost way to develop an assessment tool.^12^

Other limitations specific to the evaluation include the immediate postassessment and the fact that the evaluation tool assessed only team performance, not individual performance. The effectiveness of the curriculum would be strengthened with decay data. However, the nature of the medical student curriculum would make this very challenging to do. Regarding team performance, we feel this is an appropriate approach for a few reasons. First, critically ill children are managed by a team of providers, not individuals. Second, this is not a high-stakes (pass/fail) situation. Therefore, for this educational intervention, individual performance is less important than team performance. If individual performance evaluation is desired, we feel the assessment checklist could be easily modified to serve that role.

Additionally, there is a deficiency in the checklist as it relates to the objective concerning whether participants describe an age-appropriate differential diagnosis. The modified Delphi process did not include checklist items specific to this objective. Therefore, we are unable to comment on the effectiveness of the educational intervention as it relates to this specific objective. However, this objective is addressed during the debriefing process.

Overall, this was a successful implementation of an SBL case with a sound evaluation and assessment strategy. The simulation was so well received that we are planning to expand the simulation offerings during PRECEDE.

## Appendices


Participant Handout.docxSimulation Case.docxFaculty Guide.docxAssessment Checklist.docxCourse Evaluation.doc
