# Supplementary material for: Bronchiolitis Simulation Module in the Pediatric Preclerkship Educational Exercises (PRECEDE) Curriculum
Source: MedEdPORTAL. 2023 Jun 13;19:11318. doi: 10.15766/mep_2374-8265.11318 (PMC10261534; doi:10.15766/mep_2374-8265.11318)
Supplement: Supplementary file 1 — Participant Handout.docxSimulation Case.docxFaculty Guide.docxAssessment Checklist.docxCourse Evaluation.doc [file mep_2374-8265.11318-s001.zip › A. Participant Handout.docx]

**Appendix A: Participant Pre-Simulation Handout**

The patient is an infant with RSV bronchiolitis. The nurses have been reporting desaturations and increasing respiratory distress. The interns are at a required conference. Your senior resident asks the medical student team to go and evaluate the patient and report back.

The infant is a 38-week gestation with no preexisting medical problems.

The child came to the ED last night and, due to respiratory distress and the need for frequent suctioning, the child was admitted.

Your task is to evaluate the child, provide any necessary interventions and then report back to the faculty member or senior resident.
