# Supplementary material for: Bronchiolitis Simulation Module in the Pediatric Preclerkship Educational Exercises (PRECEDE) Curriculum
Source: MedEdPORTAL. 2023 Jun 13;19:11318. doi: 10.15766/mep_2374-8265.11318 (PMC10261534; doi:10.15766/mep_2374-8265.11318)
Supplement: Supplementary file 1 — Participant Handout.docxSimulation Case.docxFaculty Guide.docxAssessment Checklist.docxCourse Evaluation.doc [file mep_2374-8265.11318-s001.zip › B. Simulation Case.docx]

| **Appendix B: Acute Bronchiolitis Simulation**  **SIMULATION CASE TITLE: Infant Acute Bronchiolitis**  **AUTHORS: Justin M Jeffers, Amit Pahwa, Stacy Cooper, David W Cooke, Rebekah Reisig, Olivia Widger, Lauren Kahl, Edward L Bartlett, Christopher Grybauskas, Eric Balighian, Emily Frosch, W Christopher Golden**  **LEARNER AUDIENCE: Pediatric Clerkship Students** | | | | | | | |  |
| --- | --- | --- | --- | --- | --- | --- | --- | --- |
| **PATIENT NAME:**  **PATIENT AGE: Four-month-old, full-term infant**  **CHIEF COMPLAINT: Worsening respiratory distress**  **PHYSICAL SETTING: General pediatric inpatient floor/room** | | | | | | | |  |
|  | | | | | | | |  |
| **Brief Narrative Description of Case** | | This is a four-month-old full-term infant with bronchiolitis. The patient was seen in the emergency department last night and admitted for respiratory distress. Overnight, the patient did well but this morning, nurses have been reporting desaturations and increasing respiratory distress. The senior resident asks the medical students to go evaluate.  No preexisting medical problems.  In the emergency department, a beta-agonist trial did not improve symptoms.  Child admitted for supportive care.  Room is dark (lights off). Patient is bundled and in car seat (or infant swing/chair). Oxygen saturation is dropping from low 90’s to high 80’s progressively. Infant is not on O2. Pulse oximeter is on, but cardiac monitoring is not on. | | | | | |  |
| **Primary Learning Objectives** | | By the end of this simulation, participants will be able to:   1. Recognize respiratory distress as evaluated by evaluation checklist 2. Utilize the “ABC” assessment strategy and crisis resource management principles 3. Describe an age-appropriate differential diagnosis for infant respiratory distress 4. Implement a stepwise approach to treating infant respiratory distress using various oxygen delivery systems 5. Describe the indications for diagnostic tests such as imaging and labs | | | | | |  |
| **Critical Actions** | | 1. Implement sound situational awareness  - Turn on lights, remove patient from car seat/chair - Expose the patient  1. Appropriately treat respiratory distress  - Apply non-rebreather - Implement airway maneuvers (shoulder roll, head tilt, jaw thrust) - Insert appropriate airway adjunct (nasal versus oral airway adjunct) - Escalate to bag-mask ventilation when patient does not improve  1. Discuss further management  - Explore a reasonable differential diagnosis - Discuss whether or not labs and/or imaging are indicated - Call for further help appropriately – does the patient need a higher level of care? | | | | | |  |
| **Learner Preparation or Prework** | | Please see Appendix A for participant handout.  The patient is an infant with RSV bronchiolitis. The nurses have been reporting desaturations and increasing respiratory distress. The interns are at a required conference. Your senior resident asks the medical student team to go and evaluate the patient and report back.  The infant is a 38-week gestation with no preexisting medical problems.  The child came to the ED last night and, due to respiratory distress and the need for frequent suctioning, the child was admitted.  Your task is to evaluate the child, provide any necessary interventions and then report back to the faculty member or senior resident. | | | | | |  |
| Initial Presentation | | | | | | | |  |
| **Initial Vital Signs** | | Pulse: 120 beats per minute, Blood Pressure: 90/60 mmHg. Pulse Oximetry: 88% on room air and gradually decreasing. Cardiac Monitor: normal sinus rhythm but tachycardic. Respiratory Rate: 65 breaths per minute. | | | | | |  |
| **Overall Setting and Appearance** | | Team enters the room (general pediatric inpatient room) all together. Room is dark and the patient is bundled in an infant car seat or infant chair. Patient is working hard to breath with mild hypoxia as above. Patient is connected to pulse oximetry but not cardiac monitoring. | | | | | |  |
| **Standardized Participants (and Their Roles in the Room at Case Start)** | | One facilitator in the room serving as a nurse who will have all the information needed for the participants. This may be a physician as is the case with our group but would be better served by a nurse facilitator.  Upon the team entering the room, the nurse will highlight the key HPI features as below and ask the team to evaluate the patient. The nurse will answer all participant questions appropriately. They will also assist with getting the participants “unstuck” as needed. For example, if more than a few minutes goes by and participants have not turned on the lights or removed patient from the car seat, the nurse may prompt them by saying, “Do you think your exam would be easier with the lights on?”. Or “Would it be easier to examine the patient out of the car seat?”. If the participants are not escalating respiratory care, the nurse may similarly say something like, “It does not appear the non-rebreather is working. Is there anything else we could try?”. Or “I think the patient would benefit from bag-mask ventilation. What do you think?”. | | | | | |  |
| **HPI** | | Provided: This is a four-month-old full-term infant admitted last night from the emergency department with a diagnosis of RSV bronchiolitis. The patient did well overnight but this morning, has had a gradual worsening of work of breathing and the pulse oximetry seems to be trending lower.  Requiring an ask: Past history (benign except father with childhood asthma). Emergency department management (suction which helped a little and a beta agonist trial which did not help. No labs or X-Rays). The nurse has not tried anything for this acute worsening of symptoms. | | | | | |  |
| **Past Medical/Surgical History** | | **Medications** | | **Allergies** | | **Family History** | |  |
| None | | None | | None | | Father with history of childhood asthma | |  |
| **Physical Examination** | | | | | | | |  |
| **General** | | Moderate respiratory distress | | | | | |  |
| **HEENT** | | + nasal congestion and rhinorrhea | | | | | |  |
| **Neck** | | Supple, benign exam | | | | | |  |
| **Lungs** | | Tachypnea, mild retractions, occasional grunting, coarse breath sounds throughout | | | | | |  |
| **Cardiovascular** | | Mild tachycardia, normal heart sounds. Capillary refill time 2-3 seconds | | | | | |  |
| **Abdomen** | | Soft, non-tender, non-distended | | | | | |  |
| **Neurological** | | No focal findings. Cranial nerves intact. Normal movement and sensation. | | | | | |  |
| **Skin** | | Normal appearance. No rashes or bruising | | | | | |  |
| **GU** | | Normal external GU exam | | | | | |  |
| **Psychiatric** | | Besides current illness, acting appropriately and normally per parents | | | | | |  |
| Instructor Notes - Changes and CASE Branch Points | | | | | | | | |
| **Intervention / Time Point** | | | **Change in Case** | | **Additional Information** | | | |
| **Upon Room Entry –> 5 Minutes**:  Participants should turn on lights, unbundle baby, quickly assess “ABC’s”, perform initial airway maneuvers (shoulder roll, head tilt, etc.), ask for a non-rebreather, and call for help. | | | Respiratory rate slowly increases to 70. Oxygen saturations slowly decrease to mid 80’s% | | Nurse gives brief history. If Liters per minute flow not specified by the participants, nurse should ask, “How much oxygen?” Or “How high should I turn it up?” | | | |
| **5-10 Minutes:**  Participants recognize current interventions not working. They should trouble shoot current set-up while escalating care with airway adjuncts and bag-mask ventilation | | | Respiratory rate will now slowly start to decline to 30’s along with continued decline of pulse oximetry to mid-70’s%, indicating impending respiratory failure. This is averted if bag-mask ventilation occurs – pulse oximetry starts to climb to low 90’s% over 1-2 minutes with proper bag-mask ventilation. | | If participants appear stuck, nurse can offer helpful questions/statements such as:  “The patient doesn’t appear to be improving”  “Is there a way to deliver more oxygen?”  “Is there something we can do to help the work of breathing?” | | | |
| **10-15 Minutes:**  Participants should be bag-mask ventilating.  Discuss indications for further evaluation (labs, X-Rays), differential diagnosis, further patient history, and disposition | | | With proper bag-mask technique (two-person and/or airway adjunct) | | If participants appear stuck:  “Is there anyone we can call for more help?”  “Do you think any labs or medications would be helpful?” | | | |
| **15-20 Minutes:**  Help arrives (facilitator operating the manikin). Participants offer a summary of patient and interventions | | | Patient remains stable with proper bag-mask ventilation | | Scenario ends | | | |

**Ideal Scenario Flow**

Upon room entry, participants turn on the lights and recognize the abnormal vital signs on the monitor while approaching the patient. They should place patient in a position to allow appropriate evaluation (remove from car seat, unbundle, place on stretcher, etc.). While initiating care with airway positioning and oxygen supplementation, a brief relevant history should be obtained. A non-rebreather should be placed with maximum oxygen flow. A nasal airway adjunct should be considered. Within the first five minutes, this level of learner (clerkship students) should be calling for help.

As the patient’s respiratory status continues to worsen, a conversation and decision should be made rather quickly to progress to bag-mask ventilation. If there are enough participants, two-person technique should be used. Otherwise, at a minimum, airway adjuncts should be employed.

With appropriate bag-mask ventilation, the patient will improve. Once stable, further history can be obtained, and further management conversations can occur.

**Anticipated Management Mistakes**

Participants almost always take too long to remove patient from the car seat and unbundle. This is an important step because it is common for infants to be in a swing, chair, car seat, caregiver’s arms, etc. This is generally different when compared to adult patients. The purpose of this is to reinforce to participants that infants are often not in the best position for evaluation and treatment and should be placed in an optimal position quickly.

It is very common for participants to either start with regular nasal cannula or immediately ask for High Flow nasal cannula. Neither of these options is the appropriate initial response. Regular nasal cannula does not deliver enough fraction of inspired oxygen (FiO2) to improve this level of respiratory distress. High Flow nasal cannula may be what this patient ultimately needs but is rarely immediately available. A non-rebreather offers a high FiO2, which this patient needs, and bag-mask ventilation is not only an important skill for pediatricians to know but serves as a stabilizing and temporizing measure until a more definitive positive pressure set-up can arrive.

It is also common for participants to want to obtain a definitive airway with direct laryngoscopy endo-tracheal intubation. It should be reinforced that this is not a skill general pediatricians need to know, and bag-mask ventilation is the primary airway skill required.

Most participants want to obtain laboratory tests and/or a chest X-Ray. For most typical bronchiolitis patients, this is unnecessary and can potentially cause harm. There are exceptions to consider, such as a point of care glucose test if participants are worried about poor feeding.

Same goes for medication administration. Most bronchiolitis patients do not benefit from any pharmaceutical interventions. A small subset (history of atopy, strong family history of atopy) may benefit from beta-agonists. But other interventions such as steroids, hypertonic saline, and racemic epinephrine have no benefit for these patients.
