# Supplementary material for: Bronchiolitis Simulation Module in the Pediatric Preclerkship Educational Exercises (PRECEDE) Curriculum
Source: MedEdPORTAL. 2023 Jun 13;19:11318. doi: 10.15766/mep_2374-8265.11318 (PMC10261534; doi:10.15766/mep_2374-8265.11318)
Supplement: Supplementary file 1 — Participant Handout.docxSimulation Case.docxFaculty Guide.docxAssessment Checklist.docxCourse Evaluation.doc [file mep_2374-8265.11318-s001.zip › C. Faculty Guide.docx]

**Appendix C**

**FACULTY HANDOUT FOR ACUTE BRONCHIOLITIS SCENARIO**

Here is the medical Student set up for inpatient simulation

The patient is an infant with RSV bronchiolitis. The nurses have been reporting desaturations and increasing respiratory distress. The interns are at a required conference. Your senior resident asks the medical student team to go and evaluate the patient and report back.

The infant is a 38-week gestation with no preexisting medical problems.

The child came to the ED last night and, due to respiratory distress and the need for frequent suctioning, the child was admitted.

The only medication is albuterol nebulizers PRN.

Your task is to evaluate the child, provide any necessary interventions (no medications will need to be administered) and then report back to the faculty member or senior resident.

| Case Description  Infant with bronchiolitis. Nurses have been reporting desaturations and increasing respiratory distress. Senior resident asks the medical students to go evaluate.  38-week gestation. No preexisting medical problems. Child admitted for supportive care and bets-agonist nebs PRN.  Room is dark (lights off), patient is bundled and in car sear, oxygen saturation dropping from low 90’s to high 80’s progressively. Infant not on oxygen. Pulse oximeter and cardiac monitoring is on. |
| --- |

**Learner Objectives**

| 1.) Recognize respiratory distress and utilize the “ABC” assessment strategy and crisis resource management principles |
| --- |
| 2.) Describe an age-appropriate differential diagnosis for infant respiratory distress |
| 3.) Implement a stepwise approach to treating infant respiratory distress using various oxygen delivery systems |
| 4.) Describe the indications for diagnostic tests such as imaging and labs |

Initial Vital Signs

| Pulse: | 120 beats per minute |
| --- | --- |
| Blood Pressure: | 90/60 mm/Hg |
| Pulse Oximetry: | 88% gradually decreasing |
| ECG: | Normal Sinus Rhythm |
| Respiratory Rate: | 65 breaths per minute |

No intravenous access.

**SIM BABY WILL DEMONSTRATE THE FOLLOWING FINDINGS:**

Rhonchi, Crackles*,* Gurgling Rhonchi, normal circulation, normal heart tones, normal bowel sounds.

- One facilitator serves as simulation operator
- One facilitator serves as a nurse confederate
- Participants perform scenario
- Both facilitators debrief the scenario
- Participants repeat the scenario to “train to success”

| **Participants Should** | **Action** |
| --- | --- |
|  | Nurse briefly describes the patient and the reason he/she is concerned. “I am worried about the baby’s breathing and the oxygen saturation seems to be dropping.” |
| Students should turn on lights, unbundle baby, lower rails on crib. Assess ABCs quickly, identify respiratory distress, head tilt/chin lift position.  Call for additional help. | If students don’t ask for O2, nurse asks” would you like to put O2 on the baby?  If students want O2 (whether prompted or not), nurse will ask how they would like to administer (Blow by, nasal canula, face mask, non-rebreather, etc.) |
|  | Respiratory rate increases to 70. Heart rate decreases to 90. Saturations drop to mid 80’s. Blood pressure remains 90/60. |
| Students should begin bag-valve mask ventilation with 100% oxygen attached to bag and appropriate size mask. Call rapid response team and/or senior resident if not already done. | Nurse expresses worry about patient and recommends bag-mask ventilation.  Potential Prompt: “WHAT SIZE FACE MASK DO YOU WANT?” |
|  | Patient’s oxygen saturation will improve with bag-valve-mask ventilation using proper technique.  Scenario ends. |
|  | Key teaching points:  1. Rapid assessment of ABCs and exposure of patient  2. Clear leader and delegation of responsibilities  3. Recognize respiratory distress and administer oxygen quickly  4. Apply bag-valve-mask ventilation quickly when oxygen saturation is dropping despite oxygen with correct technique |

**Potential Teaching Points**

1. **Oxygen Delivery Systems**

**Low-Flow Oxygen Systems:**

The FiO2 in low flow systems (items 1-4) will vary depending on the oxygen delivery device and the patient’s respiratory/oxygenation needs.

**Abbreviations:** LPM = Liters per Minute. O2 = oxygen. FiO2 = Fraction of Inspired Oxygen. CO2 = Carbon Dioxide.

| **Device/Where Obtained** | **Liter**  **Flow**  **(LPM)** | **O2**  **Concentration**  **(FiO2)** | **Advantages** | **Disadvantages** | **Administration Guidelines** |
| --- | --- | --- | --- | --- | --- |
| 1. Nasal Cannula | 1-6  1=25%  2=29%  3=33%  4=37%  5=41%  6=45% | 22-45% | Effective for low oxygen concentrations | Will not deliver oxygen concentrations higher than 45%  Dry mucous membranes | Maximum flow is 5-6 LPM. Change to another O2 device if patient requires >5 LPM.  Humidify for liter flows >4 LPM  Use on patients with adequate tidal volume and normal vital signs |
| 2. Simple Mask | 6-10 | 25-60% | Delivers oxygen concentrations up to 60% | Tight seal is required for higher oxygen concentrations:  hot and confining  impractical long-term | A minimum of 6 LPM is required for all masks to flush expired carbon dioxide and prevent rebreathing of CO2.  Do not use humidifier and fit firmly.  Use for severe asthma, pneumonia, trauma, or severe sepsis |
| 3. Partial non-rebreather | 8-12 | 35-60% | Flaps stay open  Valves allow expired CO2 to leave the mask | Requires a tight seal  Impractical for long-term | Reservoir bag must always remain inflated  Do not use humidifier bottle  If bag collapses, increase flow rate until inflated  Ensure free expansion, no twisting, or kinks |
| 4. Non-Rebreather | 10-15 | 80-95% | Delivers the highest possible oxygen concentration without intubation  Short-term therapy | Requires a tight seal  Impractical for long-term | Reservoir bag must always remain inflated  Do not use humidifier bottle  If bag collapses, increase flow rate until inflated  Ensure free expansion, no twisting, or kinks |

**High-Flow Oxygen Systems:**

These devices (items 5-6) meet or exceed the patient’s minute volume or inspiratory demands. They deliver fixed concentration of oxygen, regardless of the inspiratory flow or breathing pattern.

| **Device/Where Obtained** | **Liter**  **Flow**  **(LPM)** | **O2**  **Concentration**  **(FiO2)** | **Advantages** | **Disadvantages** | **Administration**  **Guidelines** |
| --- | --- | --- | --- | --- | --- |
| 5. Venturi Mask | Varies (see Table 1)  Mixes a specific volume of air and oxygen | 24-60%  FiO2 is determined by the color of the venture device as stated above | Delivers highly accurate oxygen concentration for the same amount of air always enters | Requires a tight seal  Intake ports can be blocked | Accurate O2 concentration depends on oxygen liter flow and color of attached venture device.  Always use the clear plastic collar, to guarantee the oxygen concentration delivered.  Do not use a humidifier bottle |
| 6. Aerosol/Large volume Nebulizers  Trach | 10-15 | 28-100% | Administers large volumes of mist  Indicated for thick secretions | Condensation may collect in the trach. Collar or tubing | Observe for signs of overhydration, pulmonary edema, crackles  Connected to a wide corrugated tubing that receives oxygen from a jet nebulizer |

| **Table 1 Guide to colors of Venturi valves** | | |
| --- | --- | --- |
| Venturi valve | Flow rate | Oxygen delivered |
| Color | (LPM) | (%) |
| Blue | 2 | 24 |
| White | 4 | 28 |
| Yellow | 6 | 35 |
| Red | 8 | 40 |
| Green | 12 | 60 |
| Treatment with oxygen | 60% or/>101 rebreathing | 90-94 |

1. **Proper positioning of airway**
   1. Head Tilt Chin Lift UNLESS C-spine injury suspected.
   2. 2 provider – lift at angle of mandible
   3. Jaw thrust without neck extension if worried about C-spine
2. **OPTIONAL - Oral airways (OPA) / nasal airways (NPA)**
   1. OPA only for unconscious patient
   2. OPA from corner of mouth to the angle of the mandible
   3. NPA for conscious or semiconscious patient
   4. NPA from tip of nose to tragus of ear
3. **Proper Bag-Valve mask ventilation**
   1. EC hand position
   2. Chest rise necessary
   3. Listen for breath sounds
   4. Consider decompression of the stomach – cricoid pressure may minimize GI air
   5. Rate: Go slow, give time for exhalation.
4. **Recognition of respiratory failure**
   1. Changes in mental status
   2. Increased resp effort
   3. Poor air movement / poor chest rise
   4. Marked tachypnea (early)
   5. Bradypnea, apnea (late)
   6. Tachycardia (early), Bradycardia (late)
