# Supplementary material for: Bronchiolitis Simulation Module in the Pediatric Preclerkship Educational Exercises (PRECEDE) Curriculum
Source: MedEdPORTAL. 2023 Jun 13;19:11318. doi: 10.15766/mep_2374-8265.11318 (PMC10261534; doi:10.15766/mep_2374-8265.11318)
Supplement: Supplementary file 1 — Participant Handout.docxSimulation Case.docxFaculty Guide.docxAssessment Checklist.docxCourse Evaluation.doc [file mep_2374-8265.11318-s001.zip › D. Assessment Checklist.docx]

**Appendix D**

**Pediatric PRECEDE Respiratory Distress Module Assessment Tool**

Module Objectives:

1. Students will recognize respiratory distress as evaluated by evaluation checklist
2. Utilize the “ABC” assessment strategy and crisis resource management principles
3. Students will describe an age-appropriate differential diagnosis for infant respiratory distress
4. Students will implement a stepwise approach to treating infant respiratory distress using various oxygen delivery systems
5. Students will describe the indications for diagnostic tests such as imaging and labs

| **Stage of Care (time in minutes)** | **Item no.** | **Item** | **Not Done (0 points)** | **Partially or Incorrectly Done**  **(1 point)** | **Done correctly, and completely (2 points)** | **Weighting** | **Item Score** |
| --- | --- | --- | --- | --- | --- | --- | --- |
| **Situational Awareness/General Tasks (0-2)**  **Objectives: 1, 2** | 1.1 | Turns on Lights |  | - Done but took longer than 5 secs | - Done within 5 secs | 4 |  |
|  | 1.2 | Lowers bed rails |  | - Done but longer than 30 secs | - Done within 30 secs | 3 |  |
|  | 1.3 | Removes patient from car seat |  | - Done but longer than 60 secs | - Done within 60 secs | 4.5 |  |
|  | 1.4 | Removes patient gown |  | - Done but longer than 90 secs | - Done within 90 secs | 4 |  |
|  | 1.5 | Gathers brief but appropriate history |  | - Required prompting, or inappropriate details | - Appropriate and complete information gathered | 4.5 |  |
|  | 1.6 | Applies appropriate personal protection equipment (gloves for patient contact, mask if near airway) |  | - Some but not all apply personal protection equipment | - All apply equipment within 60 secs | 3.5 |  |
|  | 1.7 | Place patient on monitor (ECG, Sp02, BP) |  | - Done but longer than 90 secs | - Done within 90 secs | 5 |  |
|  | 1.8 | Clear and defined role assignment (leader, airway x 2, primary assessor, family liaison) |  | - Roles differentiated but not clearly assigned OR 3 or fewer roles assigned | - 4 or more clearly assigned and defined roles | 4 |  |
| **Initial Management (0-3)**  **Objectives: 1, 2, 3, 4** |  |  |  |  |  |  |  |
|  | 2.1 | Assess airway and breathing via clear effort such as auscultation, verbal recognition of respiratory vital signs, etc. |  | - Assess one or the other, or not timely | - Done within 30 secs | 5 |  |
|  | 2.2 | Recognizes respiratory distress via verbalization or clear attempt at intervention |  | - Done but longer than 90 secs | - Done within 90 secs | 5 |  |
|  | 2.3 | Attempts airway opening maneuvers – Head tilt, jaw thrust, chin lift, or shoulder roll |  | - Only does 1 or multiple but incorrectly done, or not timely | - Does multiple correctly within 90 secs | 5 |  |
|  | 2.4 | Apply O2 |  | - Nasal cannula > 6L or NRB <10L or not timely. | - 100% NRB at >10L within 120 secs or escalated approach within 180 secs | 5 |  |
|  | 2.5 | Assesses circulation – HR, BP, access, CRT |  | - Done but not all measures or longer than 120 secs | - All measures done within 120 secs | 5 |  |
|  | 2.6 | Utilizes appropriate team-based communication – closed loop within team, appropriate and timely family communication, frequent verbal reassessment/summary |  | - Rarely or sometimes | - Usually or always | 4 |  |
| **Escalation of Care (2-10)**  **Objectives: 1, 2, 3, 4** |  |  |  |  |  |  |  |
|  | 3.1 | Recognizes initial interventions are not working |  | - Either verbalize OR intervene OR >30 secs from placing O2 | - Verbalize AND intervene within 30 secs of placing O2 | 4.5 |  |
|  | 3.2 | Places oral and/or nasal airway |  | - Placed but did not measure for size OR longer than 90 secs | - Done with proper size AND within 60 secs | 3.5 |  |
|  | 3.3 | Initiates BMV using proper technique (EC or two-person method) |  | - Done but improper technique OR longer than 60 secs | - Done properly within 60 secs of recognizing need for further intervention | 4.5 |  |
|  | 3.4 | Calls for more help |  |  | - Verbalized at any point during scenario | 4.5 |  |
|  | 3.5 | Reassess after each intervention |  | - Reassess after 2 or fewer interventions | - Reassess after 3 or greater interventions | 4.5 |  |

Abbreviations: ECG indicates electro-cardiogram; SpO2, oxygen saturation; BP, blood pressure; secs, seconds; NRB, non-rebreather; BMV, bag mask ventilation; CRT, capillary refill time; L, liter; O2, Oxygen

Time in seconds from entering room to effective BMV:__________

Total score (out of 166): ______

Date:

Pre-Intervention:____

Post-Intervention:____
