# Supplementary material for: Bronchiolitis Simulation Module in the Pediatric Preclerkship Educational Exercises (PRECEDE) Curriculum
Source: MedEdPORTAL. 2023 Jun 13;19:11318. doi: 10.15766/mep_2374-8265.11318 (PMC10261534; doi:10.15766/mep_2374-8265.11318)
Supplement: Supplementary file 1 — Participant Handout.docxSimulation Case.docxFaculty Guide.docxAssessment Checklist.docxCourse Evaluation.doc [file mep_2374-8265.11318-s001.zip › E. Course Evaluation.doc]

**Appendix E**

**PRECLERKSHIP EDUCATIONAL EXERCISES (PRECEDE) Course Evaluation Tool**

**Clerkship:** PEDIATRICS

**Session Topic / Title:** Acute Bronchiolitis Module

**Small Group Leaders (if applicable):**

Please provide feedback on this PRECEDE session and the session instructors

SD: Strongly Disagree	D: Disagree	A: Agree	SA: Strongly Agree

|  | **Session** | SD | D | A | SA |
| --- | --- | --- | --- | --- | --- |
| 1 | This session met the stated objectives |  |  |  |  |
| 2 | This session increased my SKILLS related to this topic |  |  |  |  |
| 3 | Having had this session will improve my ability to function as a clinical clerk on the wards |  |  |  |  |
| 4 | This session was educationally valuable |  |  |  |  |
| 5 | Please rate this session overall | **POOR** | **FAIR** | **GOOD** | **EXCELLENT** |
|  | **Lecturers / Small Group Leaders:** |  |  |  |  |
| 6 | The instructors provided a supportive learning environment |  |  |  |  |
| 7 | Active student participation was encouraged |  |  |  |  |
| 8 | The instructors enabled me to accomplish the learning objectives for the session |  |  |  |  |
| 9 | The instructors’ teaching methods stimulated my own critical thinking |  |  |  |  |
| 10 | Rate the instructors / facilitators overall | **POOR** | **FAIR** | **GOOD** | **EXCELLENT** |

Do you have any recommendations to improve this session? If so, please provide.
